# Antiretroviral resistance following immunological monitoring in a resource-limited setting of western India: A cross-sectional study

**DOI:** 10.1371/journal.pone.0181889

**Published:** 2017-08-01

**Authors:** Santosh K. Karade, Smita S. Kulkarni, Manisha V. Ghate, Ajit A. Patil, Rajkumar Londhe, Sonali P. Salvi, Dileep B. Kadam, Rajneesh K. Joshi, Bharat B. Rewari, Raman R. Gangakhedkar

**Affiliations:** 1 HIV Drug Resistance Laboratory, National AIDS Research Institute (ICMR), Pune, India; 2 Department of Microbiology, Armed Forces Medical College, Pune, India; 3 Department of Virology, National AIDS Research Institute (ICMR), Pune, India; 4 Department of Clinical Sciences, National AIDS Research Institute (ICMR), Pune, India; 5 Department of Medicine, BJ Medical College and Sasoon General Hospital, Pune, India; 6 Department of Epidemiology and Biostatistics, National AIDS Research Institute (ICMR), Pune, India; 7 Department of Community Medicine, Armed Forces Medical college, Pune, India; 8 Department of AIDS Control, National AIDS Control Organization, New Delhi, India; Hôpital Bichat-Claude Bernard, FRANCE

## Abstract

**Background:**

The free antiretroviral therapy (ART) program in India still relies on the clinico-immunological monitoring for diagnosis of treatment failure. As the nucleoside reverse transcriptase inhibitor (NRTI) backbone is shared in first- and second-line regimens, accumulation of drug resistant mutations (DRMs) can compromise the efficacy of NRTI. This study was undertaken to describe the pattern of HIV DRMs following immunological monitoring and investigate its impact on the cycling of NRTI between first- and second-line ART.

**Methods and findings:**

This cross-sectional study was performed at a state-sponsored ART clinic of Pune city in western India between January and June 2016. Consecutive adults receiving first-line ART with immunological failure (IF) were recruited for plasma viral load (PVL) estimation. Randomly selected 80 participants with PVL >1000 copies/mL underwent HIV drug resistance genotyping. Of these, 75 plasma sample were successfully genotyped. The median CD4 count and duration of ART at the time of failure were 98 (IQR: 61.60–153.50) cells/μL and 4.62 (IQR: 3.17–6.15) years, respectively. The prevalence of NRTI, non-NRTI, and major protease inhibitor resistance mutations were 89.30%, 96%, and 1.33%, respectively. Following first-line failure, sequences from 56.67% of individuals indicated low- to high-level resistance to all available NRTI. The proportion of sequences with ≥2 thymidine analogue mutations (TAMs) and ≥3 TAMs were 62.12% and 39.39%, respectively. An average of 1.98 TAMs per sequence were observed following IF as compared to 0.37 TAMs per sequence following targeted PVL monitoring at 12 months of ART from a prior study; this difference was significant (p<0.001).

**Conclusion:**

The option of cycling of NRTI analogues between first- and second-line regimens would no longer be effective if individuals are followed-up by immunological monitoring due to accumulation of mutations. Introduction of routine PVL monitoring is a priority for the long-term sustainability of free ART program in India.

## Introduction

In India, the free antiretroviral therapy (ART) program has scaled up considerably from eight ART centers in 2004 to over 519 by 2015 [1]. In last decade, successful implementation of the National AIDS Control Program has contributed to a decline in the estimated adult HIV prevalence, from 0.34% (0.25–0.43%) in 2007 to 0.26% (0.22%-0.32%) in 2015 [2]. With the rapid rollout of ART, the consequential emergence and propagation of drug resistance is inevitable. Prior studies from India have reported variable HIV drug resistance (HIVDR) prevalence (93.80%-100%) following clinico-immunological failure (IF) of first line ART [3–7]. The prevalence of resistance mutations were lower following targeted plasma viral load (PVL) testing at 12 months of ART [8–10]. In order to make midcourse corrections in the decade-old program, periodic monitoring of HIVDR is essential.

As per national guidelines, first-line ART in adults consists of two nucleoside analogue reverse transcriptase inhibitors (NRTI) and a non-nucleoside reverse transcriptase inhibitor (NNRTI) [11]. Following failure of the first-line regimen, the second-line regimen consists of ritonavir-boosted protease inhibitors (rPI) supported by two NRTI, one of which should be new [12]. The PVL monitoring is a sensitive test and is more likely to indicate treatment failure earlier compared to clinical or immunological monitoring [13]. The free ART program in India continues to rely on the WHO criteria for the detection of clinical and immunological failure in the diagnosis of first-line ART failure [14]. As an NRTI backbone is shared in first- and second-line regimens, accumulation of drug resistance mutations (DRMs) can compromise the efficacy of NRTI. The primary objective of this study was to describe the pattern of HIV-DRMs following immunological monitoring in a state-sponsored ART clinic. Secondarily, we also investigated the impact of the accumulation of DRMs on the therapeutic options for NRTIs to be used in second-line ART.

## Materials and methods

### Study design and sample collection

The study was carried out at the state-sponsored "Centre of Excellence" ART clinic of Pune city in western India between January and June 2016. In this cross-sectional study, we recruited consecutive adults with features of IF who were referred for confirmation of failure by PVL estimation. The eligibility criteria were individuals with age >18 years who were initiated on first-line ART as per national guidelines and had an adherence rate of over 95% in the past three months. IF was defined as per WHO definition of clinical and immunological failure, wherein, fall in CD4 cell count to baseline or below and persistent CD4 cell count of less than 100 cells/μL were considered [14]. Estimation of PVL was performed using Abbott RealTime HIV-1 *m2000rt* system and virological failure was defined as a single HIV-1 PVL of more than 1000 copies/mL [14]. Five milliliters of whole blood from each individual was collected in EDTA vacutainer tubes and transported to the laboratory for PVL estimation and HIVDR genotyping. For individuals undergoing resistance testing, the details of adherence, socio-demographic profile, prior antiretroviral (ARV) drug exposure during first-line ART, drug substitutions, co-infections, and previous CD4 cell counts were noted from the individual's medical records. Drug adherence was calculated by averaging the medication possession ratio of the recent 3 months. Substitution was defined as replacement of any first-line ARV (usually by an agent of same class) due to toxicity or drug interaction.

### HIV drug resistance genotyping

Randomly selected 80 subjects with PVL > 1000 copies/ml underwent HIV drug resistance (DR) genotyping at WHO accreditated HIV drug resistance genotyping laboratory of the National AIDS Research Institute (ICMR), Pune, India. Genotyping of complete protease (PR; codon 1 to 99) and partial reverse transcriptase (RT; codon 1 to 256) region of the *pol* gene was performed by a previously validated method [15]. Following sequencing, resistance pattern was determined by Stanford University genotype resistance interpretation algorithm, HIVdb version 8.3. The thymidine analogue mutations (TAMs) considered for analysis were, TAM-1, which includes M41L, L210W and T215Y; and TAM-2, which includes D67N, K70R, T215F and K219Q/E. Other clinically relevant NRTI mutations included were M184V/I, K65R, K70E, L74V/I and Y115F. Any accessory mutation present in excess of 10% were also mentioned. The NNRTI mutations considered were L100I, K101E/P, K103N/S, V106A/M, E138A/G/K/Q, Y181C/I/V, Y188L/C/H, G190A/S/E and M230L. The predicted susceptibility of an ARV was decided by adding penalty scores associated with each DRMs in a given sequence (https://hivdb.stanford.edu/DR/asi/releaseNotes/#hivdb_ mutationpenaltyHeader). The degree of resistance was categorized into 5 levels based on total penalty score; susceptible (0–9), potential low-level resistance (10–14), low-level resistance (15–29), intermediate resistance (30–59) and high-level resistance (>60). For analysis purpose, two level category was created with a total score of 0–14 was considered as susceptible and a score of >15 was considered resistant. Phylogenetic tree of partial *pol* gene study sequences along with reference sequences was constructed by the maximum likelihood method based on general time reversible model, with MEGA 6.0 software. The reliability of the branching orders was tested by bootstrap analysis of 1000 replicates.

### Statistical analysis

Characteristics of the study participants were summarized by median and interquartile range (IQR) for continuous variables and by proportion for categorical variables. The proportion of sequences with thymidine analogue mutations (TAMs; M41L, D67N, K70R, L210W, T215Y/F, and K219Q/E) from the individuals with immunological failure in present study was compared with the sequences from a virological failure (VF) group of prior study by Karade et al. [9]. In this study, virological suppression was assessed in 847 participants at 12 ± 2 months of ART initiation and HIV drug resistance genotyping was performed in 80 individuals with virological failure (VF) [9]. Differences between these two groups were compared using the Student’s t-test for continuous variables and by Pearson’s Chi-square test for categorical variables.

### Ethical consideration

The study was approved by Ethics committee of National AIDS Research Institute and the participants were enrolled after obtaining written informed consent.

## Results

**Fig 1:**
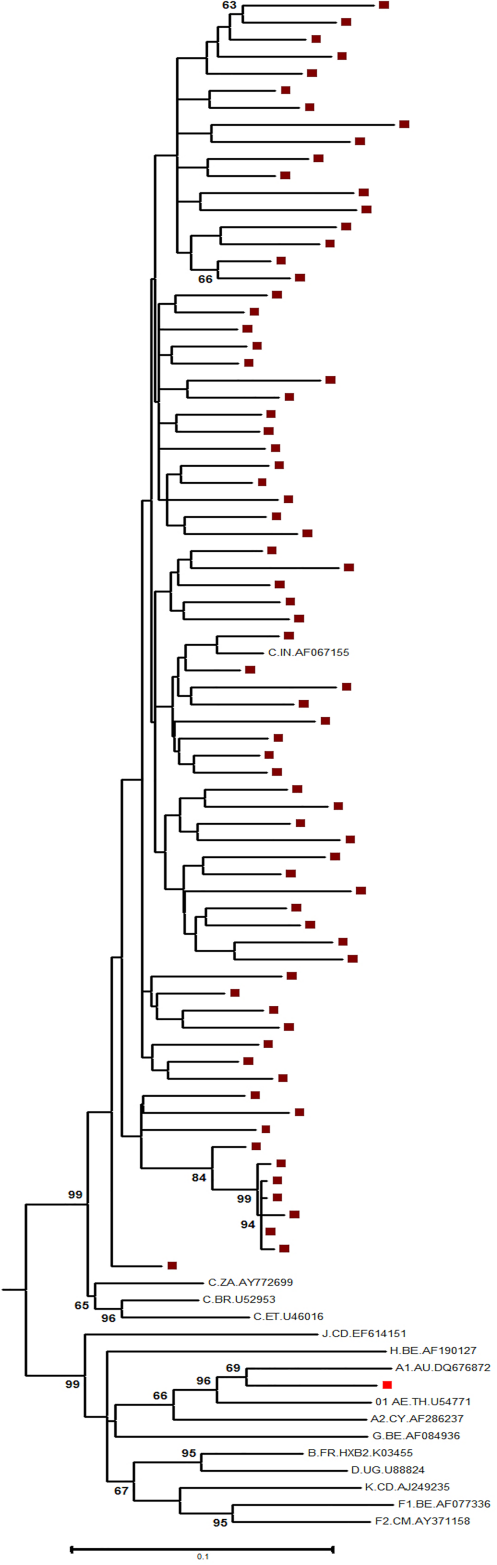
Phylogenetic tree. The phylogenetic tree shows the relationship of 75 partial *pol* gene study sequences with reference sequences. The study sequences are highlighted by solid circles.

A total of 387 individuals on first-line ART with evidence of immunological failure (IF) reported for PVL testing. Eighty plasma samples, with PVL > 1000 copies/mL were randomly chosen for drug resistance genotyping. Of these, 75 samples, including those from 50 males, were successfully genotyped. The demographic characteristics of the participants at the time of first-line ART failure are summarized in **Table 1.** A total of 56% of individuals with IF underwent treatment switches in the past due to the reasons like adverse drug reactions, drug stock out, and change in the national policy. Importantly, 21 out of 33 patients on TDF based failing regimen had prior exposure to AZT or d4T in past. The proportion of individuals with failure of zidovudine (AZT)- and tenofovir (TDF)-based regimens were 56% and 44%, respectively. Phylogenetic analysis of the partial *pol* gene sequences showed that 74 sequences clustered with the reference sequence of the Indian HIV-1 subtype C virus; one isolate clustered with subtype A1 **(Fig 1)**. The comparison of demographic features of IF group and VF group is shown in **S1 Table**.

**Table 1:** Demographic details of the 75 study participants with failure of first-line ART.

Characteristics of participants	Total, n = 75
**Gender, Male, n (%)**	50 (66.67)
**Patient Age, Median (IQR), in years**	39 (34–43)
18–30, n(%)	12 (16)
31–40	32 (46.67)
41 and above	31 (41.33)
**Marital status, n(%)**	
Married or Living-in partner	52 (69.33)
Divorced/separated/Widow	17 (22.67)
Unmarried	6 (8)
**Educational status, n(%)**	
Illiterate	16 (21.33)
Primary School	16 (21.33)
Secondary school	33 (44)
College and above	10 (13.33)
**Predominant mode of transmission**	
Heterosexual, n(%)	70 (93.33)
**Past history of tuberculosis**	25 (33.33)
**CD4 (cells/μL) at failure, Median (IQR)**	98 (61.60–153.50)
less than 100, n(%)	39 (52)
100–200	29 (38.67)
201–300	4 (5.33)
more than 300	3 (4)
**Median (IQR) VL at failure (log**_**10**_ **copies/ml)**	4.87 (4.47–5.24)
3–3.9, n(%)	8 (10.67
4–4.9	34 (45.33)
>5	33 (44)
**Failing ART regimen, n(%)**	
AZT+3TC+NVP	38 (50.67)
AZT+3TC+EFV	4 (5.33)
TDF+3TC+NVP	2 (2.67)
TDF+3TC+EFV	31 (41.33)
**First-line ART switch, n(%)**	
ART regimen never switched	33 (44)
1 to 2 switches in regimen	35 (46.67)
3 or more switches in regimen	7 (9.33)
**Duration of ART (yrs) Median (IQR)**	4.62 (3.17–6.15)
≤ 2 years	10 (13.33)
2.1–6 yrs	42 (55.99)
> 6 yrs	23 (30.67)

(Abbreviations; ART—antiretroviral therapy, VL—plasma viral load, IQR—interquartile range, AZT—zidovudine, 3TC—lamivudine, TDF—tenofovir disoproxil fumarate, NVP—nevirapine and EFV—efavirenz.)

**Fig 2:**
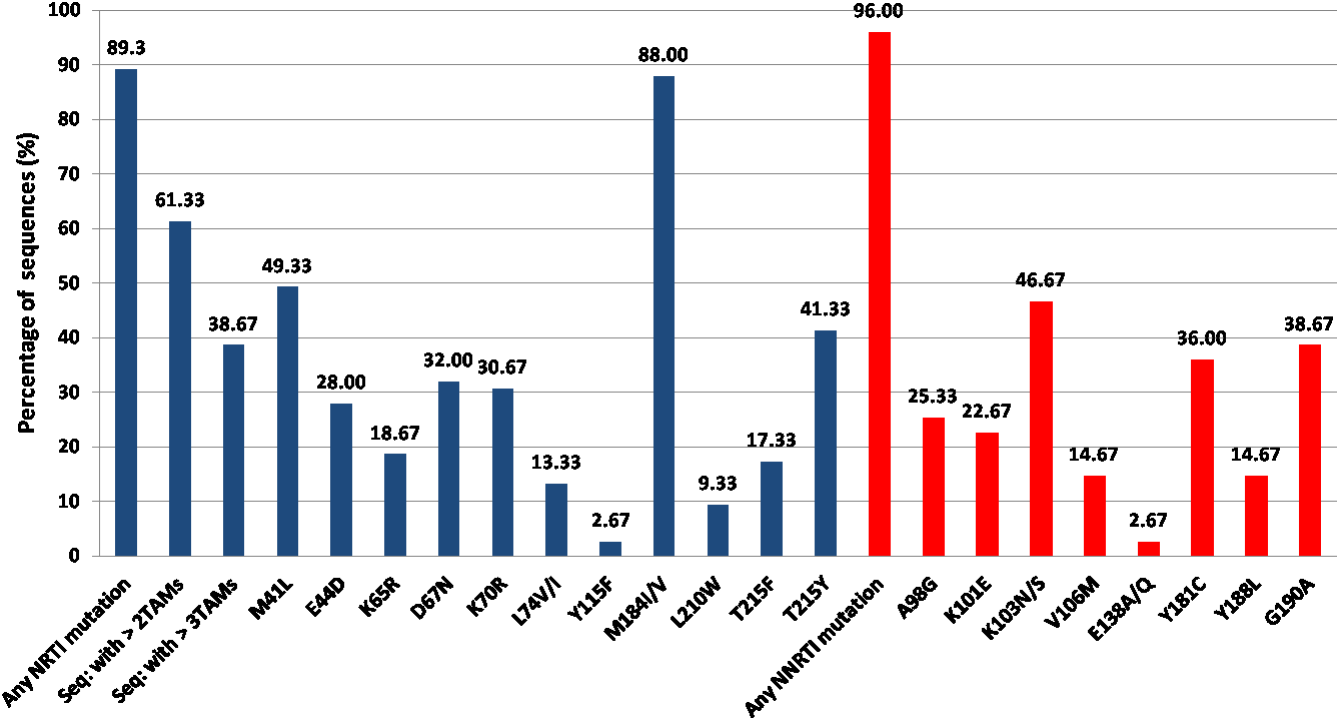
HIV drug resistance pattern. Pattern of NRTI and NNRTI drug resistance mutations following immunological failure. Abbreviations: NRTI—nucleoside/nucleotide analogue reverse transcriptase inhibitor, NNRTI—non-nucleoside reverse transcriptase inhibitor.

The prevalence of NRTI, NNRTI, and major protease inhibitor (PI) resistance mutations following immunological monitoring were 89.30%, 96%, and 1.33%, respectively. M184V (88%) and K103N (46.67%) were the predominant NRTI and NNRTI resistance mutations, respectively. M41L (49.33%) and T215Y (41.30%) were the most common TAMs. The clinically relevant NRTI and NNRTI mutations are shown in **Fig 2**. A total of 72% of the sequences indicated the presence of TAMs, with an average of 1.98 TAMs per sequence. The differences in the proportion of sequences with TAM-1 (57.33%) and TAM-2 (49.33%) mutations were not significant **(S2 Table)**. The number of sequences with ≥2 and ≥3 TAMs were 61.3% and 38.67%, respectively. The average TAMs in patients failing AZT based regimen (2.14 TAMs/sequence) was higher as compared to those on TDF based regimen (1.78 TAMs/sequence), however this difference was not significant (p = 0.34). One sequence showed the presence a insertion at codon 69 (T69 insertion) of reverse transcriptase (RT) while another sequence carried the Q151M complex mutations; each of these mutations are known to confer pan-NRTI drug resistance. In addition, another sequence harbored an amino acid deletion at codon 67 of RT. Major PI resistance mutations (M46I and N88S) was observed in a single sequence, whereas four sequences showed accessory mutations Q58E, G73C, T74P, and L10F.

**Fig 3:**
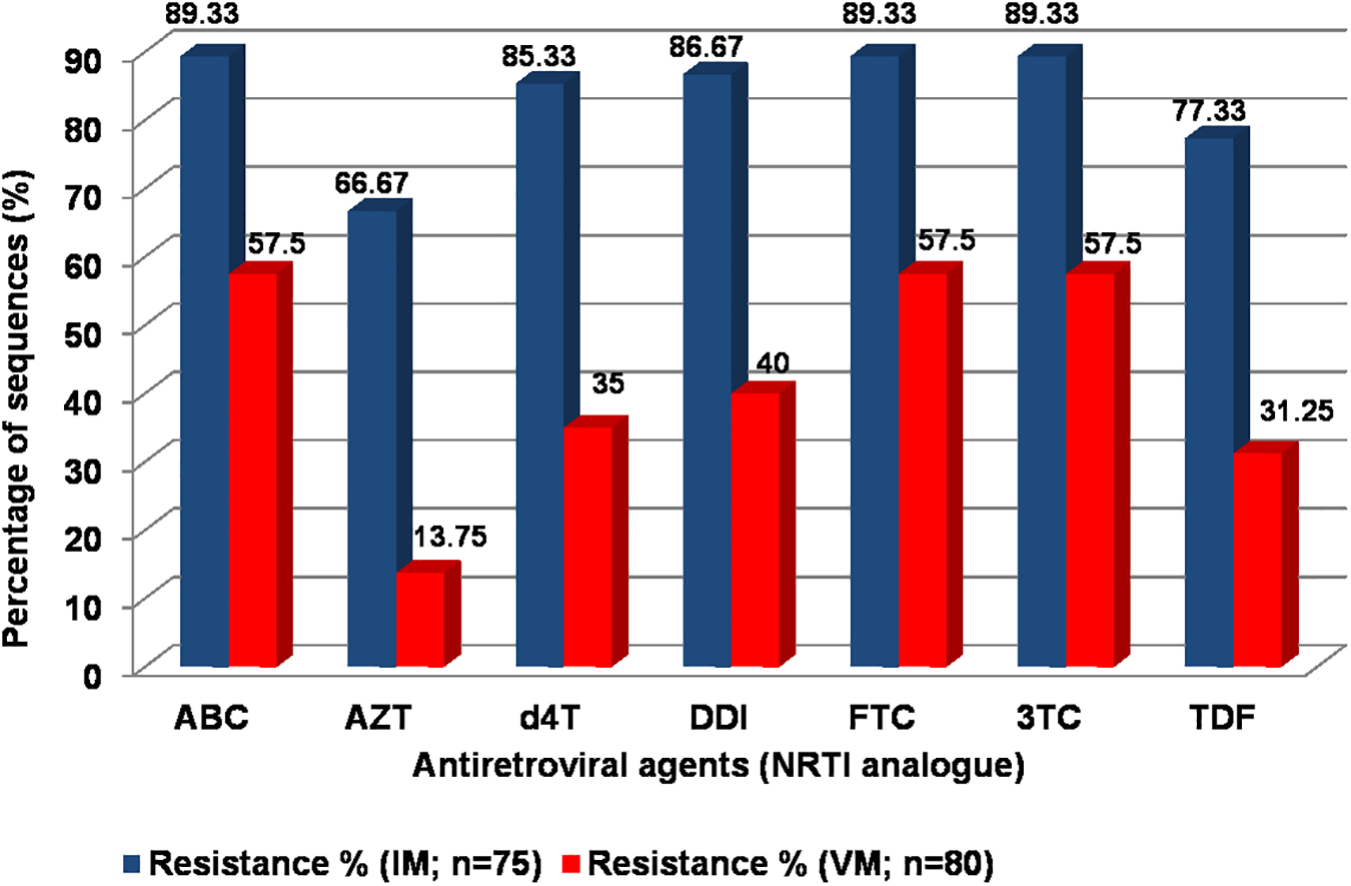
Predicted susceptibility to NRTI. The predicted susceptibility to NRTI following immunological monitoring (IM; n = 75) is compared with those from individuals with virological failure detected by targeted viral load monitoring at 12 months of ART (VM; n = 80).

Following first-line ART failure, the susceptibility to various NRTI was ascertained for cycling in second-line ART. Based on the Stanford database HIVDR scoring system, low- to high-levels of resistance to zidovudine (AZT) and tenofovir (TDF) was seen in 65.33% and 49.33% of sequences, respectively. Predicted susceptibility to NRTI in sequences from the present study were compared with our prior study, wherein first-line ART failure was diagnosed by virological monitoring at 12 months of ART [9]. The proportion of sequences with low- to high-level resistance to TDF and AZT were significantly higher following immunological monitoring (p < 0.005) **(Fig 3).**

## Discussion

In this study, we report the drug resistance outcomes in individuals with failure of first-line ART, diagnosed as per the current national guidelines. The decade-old National AIDS Control Program still relies on immunological monitoring, wherein six-monthly CD4 cell count measurements are performed, while PVL estimation is only reserved for confirmation of first-line ART failure [11]. In a prior study conducted in India, evaluation of the immunological criteria for detecting virological failure in individuals receiving first-line ART indicated a sensitivity and specificity of 22.80% and 94.60%, respectively [16]. Delayed diagnosis of failure not only causes accumulation of DRMs but also increases cross-resistance to NRTI analogues. In a study among Malawians with failure of first-line ART, Hosseinipour et al. reported compromised activity of NRTI agents in 17% of the patients [17]. A prior study from southern India reported TAMs in 53.40% of the subjects following first-line immunological failure with predominance of the M41L (40%) mutation [18]. In our study, 70% of the sequences indicated presence of TAMs, with 38.66% showing more than 3 TAMs per sequence (S2 **Table**). Furthermore, 56.67% of the sequences showed intermediate- to high-level resistance to both TDF and AZT, rendering cycling of NRTI option between first- and second-line ART ineffective. Therefore, in absence of PVL and HIVDR testing, over half of the individuals with immunological failure will be exposed to non-productive NRTI backbone in second-line ART.

The evolution of TAMs can be limited by early diagnosis of treatment failure. Reynolds et al. compared the HIVDR pattern between a group monitored by 6-monthly PVL testing (VLM group) and another, monitored by 6-monthly CD4 cell count (IM group) [19]. The study reported significantly higher rates of TAMs in the IM group (49%) than in the VLM group (5%) [19]. Targeted PVL testing at 12 months of ART may be a feasible option for low- and middle-income countries. In a study involving 142 subjects with virological failure from 6 sub-Saharan African countries, the prevalence of TAMs at the end of one year of ART was just 8.50% [20]. Previously, we reported the presence of TAMs in 17.50% sequences following virological failure at 12 months of ART [9]. The predicted susceptibility of NRTI analogues following IM, were significantly lower than that following targeted virological monitoring at 12 months of ART due to accumulation of TAMs.

In the EuroSIDA prospective observational cohort, the rate of accumulation of TAMs in subjects who continued receiving failing regimens was relatively lower (on average, 1 additional TAM accumulated every 4.3 years of exposure to the failing regimen) [21]. Unlike in a research-study setting, where there is dedicated staff and infrastructure, in resource-limited settings, TAM accumulation could be underestimated due to delayed ART initiation, inefficient monitoring, and episodes of ARV stock-out. A primarily subtype-C driven HIV epidemic, lack of pre-treatment genotypic resistance testing facility and suboptimal adherence to ART might further contribute to the adverse resistance outcome in Indian setting [22–24]. A prior study has reported higher frequency of DRMs in HIV-1 subtype C and CRF01_AE, than in subtype B infected people, following first-line ART failure [25].

Evolution of resistance is a continuous process. The prohibitive cost limits inclusion of HIV drug resistance testing into the National AIDS Control Program. The clinico-immunological monitoring identification of treatment-failure results in the development of complex DRMs, thereby compromising the NRTI backbone in second-line ART [17, 26]. Under such circumstances, one can use ritonavir boosted darunavir and/or integrase strand transfer inhibitors (INSTI), such as raltegravir and dolutegravir to support PI. Neither of them may be a feasible option for second-line ART in resource-limited setting. The choice of any of these ARV drugs will also affect options for third-line ART. At the time of failure, the median CD4 count of 98 cells/μL (IQR: 61.60–153.50) and viral load of 4.87 (IQR: 4.47–5.24) log_10_ copies/mL is indicative of longstanding failure in our study. Lower CD4 cell count and high PVL not only increases the risk of opportunistic infections but also enhances the transmission risk to sexual partners. Therefore, viral load monitoring is essential for early identification of failure. Of note, our study results are limited by the cross-sectional study design and small sample size. In addition, pre-treatment HIVDR reports for the study participants were not available and were presumed to be below 5% [27, 28]. Furthermore, even though we performed PVL and HIVDR testing on same plasma sample of all patients, a delay of 2 weeks was experienced from the time of detection of IF to PVL testing, as many of our patients are referred to us from distant clinics.

To conclude, we report extensive HIV DRMs in patients failing first-line ART. Thus, periodic surveillance of HIVDR at the national level is essential to guide the program. The option of cycling of NRTIs between first- and second-line regimens may no longer be effective if individuals are followed-up by immunological monitoring. Finally, access to periodic PVL monitoring and HIVDR testing is a necessity for long-term preservation of the efficacy of current therapeutic options.

## Supporting information

S1 Table Study group comparison. Comparison of demographic and clinical characteristics of virological monitoring group (VF) and immunological monitoring group (IF).
(DOCX)

S2 Table Thymidine analogue mutation (TAM) pattern. Comparison of TAMs in sequences from immunological failure group (IM group) with those retrieved from individuals with virological failure at 12 months of ART (VM group).
(DOCX)
