# Supplementary material for: Antiretroviral resistance following immunological monitoring in a resource-limited setting of western India: A cross-sectional study
Source: PLoS One. 2017 Aug 1;12(8):e0181889. doi: 10.1371/journal.pone.0181889 (PMC5538665; doi:10.1371/journal.pone.0181889)
Supplement: S1 Table — Comparison of demographic and clinical characteristics of virological monitoring group (VF) and immunological monitoring group (IF). (DOCX) [file pone.0181889.s001.docx]

| **Characteristics of participants** | **VF, n=104** | **IF, n=75** |
| --- | --- | --- |
| **Sex, Male - n (%)** | 54 (51.9) | 50 (66.67) |
| **Patient Age, Median (IQR), in years** | 36 (31-43) | 39 (34 - 43) |
| 18 - 30, n(%) | 24 (23.1) | 12 (16) |
| 31 - 40 | 44 (42.30) | 32 (46.67) |
| 41 and above | 36 (34.60) | 31 (41.33) |
| **Predominant mode of HIV infection** |  |  |
| Heterosexual contact | 94 (90.40) | 70 (93.33) |
| **CD4 (cells/µL) count at failure,**  **Median (IQR)** | 240 (140-355) | 98 (62 - 154) |
| less than 100, n(%) | 17 (16.3) | 39 (52) |
| 100 - 200 | 29 (27.9) | 29 (38.67) |
| 201 - 300 | 24 (23.1) | 4 (5.33) |
| more than 300 | 34 (32.7) | 3 (4) |
| **Mean Duration of ART** | 12 months | 4.73 years |
| **Failing ART regimen, n(%)** |  |  |
| AZT+3TC+NVP | 49 (47.1) | 38 (50.67) |
| AZT+3TC+EFV | 12 (11.5) | 4 (5.33) |
| TDF+3TC+NVP | 32 (30.8) | 2 (2.67) |
| TDF+3TC+EFV | 11 (10.6) | 31 (41.33) |
| **Antiretroviral substitution, n(%)** |  |  |
| No substitution | 65 (62.50) | 33 (44) |
| NRTI substitution | 21 (20.19) | 37 (49.33) |
| NNRTI substitution | 22 (21.15) | 35 (46.66) |
| **Adherence to regimen** |  |  |
| Low adherence, < 95%, n(%) | 40 (38.5) | nil |
| **Past history of tuberculosis** | 21 (20.2) | 25 (33.33) |
| **Median VL at failure (log_10_ copies/ml)** | 4.52 (3.7-5.2) | 4.87 (4.47 - 5.24) |
| 3 - 3.9, n(%) | 36 (34.6) | 8 (10.67 |
| 4 - 4.9 | 37 (35.6) | 34 (45.33) |
| >5 | 31 (29.8) | 33 (44) |

**S1 Table.** Comparison of demographic and clinical characteristics of virological monitoring group (VF) and immunological monitoring group (IF).
