# Supplementary material for: Antiretroviral resistance following immunological monitoring in a resource-limited setting of western India: A cross-sectional study
Source: PLoS One. 2017 Aug 1;12(8):e0181889. doi: 10.1371/journal.pone.0181889 (PMC5538665; doi:10.1371/journal.pone.0181889)
Supplement: S2 Table — Comparison of TAMs in sequences from immunological failure group (IM group) with those retrieved from individuals with virological failure at 12 months of ART (VM group). (DOCX) [file pone.0181889.s002.docx]

**S2 Table.** **Thymidine analogue mutation (TAM) pattern**. Comparison of TAMs in sequences from immunological failure group (IM group) with those retrieved from individuals with virological failure at 12 months of ART (VM group).

| **Study parameter** | **IM Group**  **n=75** | **VM Group**  **n=80** | **Is the difference between groups significant ?** | **Statistical test** |
| --- | --- | --- | --- | --- |
| Sequences with any TAM | 54/75 | 14/80 | Yes, p < 0.0001 | Chi-square |
| Sequences with any TAM -1 | 43/75 | 8/80 | Yes, p < 0.0001 | Chi-square |
| Sequences with any TAM -2 | 37/75 | 10/80 | Yes, p < 0.0001 | Chi-square |
| Sequences with >2 TAM | 46/75 | 8/80 | Yes, p < 0.0001 | Chi-square |
| Sequences with >3 TAM | 29/75 | 4/80 | Yes, p < 0.0001 | Chi-square |
| Average TAM per sequence | 1.98 | 0.375 | Yes, p < 0.0001 | T test |
| Mean CD4 cells/µl | 115.96 | 248.27 | Yes, p <0.0001 | T test |
| Mean viral load (copies/ml) | 193728.8 | 225667.2 | No, p = 0.63 | T test |
